# Personalized trajectory inference framework integrating driving behavior recognition and temporal dependency learning

**DOI:** 10.1371/journal.pone.0326937

**Published:** 2025-07-10

**Authors:** Jinhao Yang, Junwen Cao, Mingyu Fang

**Affiliations:** School of Science, Minzu University of China, Beijing, China; Tongji University, CHINA

## Abstract

This study proposes a Driving style-Tri Channel Trajectory Model (DS-TCTM) to enhance vehicle trajectory prediction accuracy and driving safety. The framework operates through three rigorously designed stages: (1)Data preprocessing involving kinematics feature extraction, (2)Driving style recognition utilizing acceleration variation rate and average time headway combined with K-Means++ traffic density clustering and K-neighbor Gaussian mixture model (K-GMM) analysis to classify driving behaviors into conservative, moderate, and radical categories, and (3)Personalized trajectory prediction employing a multi-level neural architecture with dedicated sub-networks for distinct driving styles. Experimental evaluations demonstrate DS-TCTM’s superior performance across multiple dimensions. The model achieves a mean RMSE of 4.46 and NLL of 3.89 across varying prediction horizons, with 35.8% error reduction attained after 100 hyperparameter optimization iterations. Comparative analysis with baseline models (LSTM, Social-LSTM, Social-Velocity-LSTM, Convolutional-Social-LSTM) reveals particularly enhanced accuracy in long-term predictions. These results confirm DS-TCTM’s effectiveness in capturing driving style impacts on trajectory patterns, providing reliable prediction enhancements for vehicle safety systems. This methodology advances personalized trajectory modeling with practical intelligent transportation applications.

## Introduction

Traffic safety remains a global critical issue. According to World Health Organization statistics [1], approximately 1.35 million fatalities and tens of millions of injuries occur annually due to traffic accidents, resulting in economic losses accounting for nearly 3% of global GDP. The advancement of autonomous driving technology offers new prospects for safety improvements, where vehicle trajectory prediction plays a pivotal role in enhancing system reliability.

In trajectory prediction research, physics-based approaches initially dominated the field. Qiao *et al*. [2] and Wiest *et al*. [3] integrated Gaussian mixture models with kinematic characteristics, achieving precise predictions in simple scenarios but demonstrating limited adaptability in complex environments. Gao *et al*. [4] enhanced motion trend capture through time-series analysis, yet struggled with abrupt behavior prediction. Xu [5] and Xie *et al*. [6] combined physical models with maneuvering behaviors to improve environmental adaptability at the cost of computational complexity. Li *et al*.’s [7] real-time planner improved dynamic environment responsiveness but required trade-offs between real-time performance and accuracy. While physics-based methods excel in controlled scenarios, their heavy reliance on precise physical inputs limits effectiveness in dynamic traffic environments. This constraint has driven researchers toward probabilistic modeling to better handle uncertainties. Zong *et al*. [8] developed a dual-layer hidden Markov model for driver intention recognition, improving prediction accuracy through intention variation analysis, albeit with compromised real-time performance. Xie *et al*. [9] employed dynamic Bayesian networks with distributed genetic algorithms to construct driving behavior perception models, requiring substantial computational resources for training. Hu *et al*. [10] applied decision trees to predict lane-change avoidance behaviors but faced limitations in complex behavioral scenarios. Schlechtriemen *et al*. [11] proposed probabilistic regression for rare event prediction (e.g., lane-change timing), though data scarcity impacts model training.

Recent advancements leverage data-driven approaches to overcome traditional limitations. Chandra *et al*.’s [12] Traphic model captures traffic interactions through weighted deep learning mechanisms. Li *et al*. [13] introduced Grip for graph-structured interaction modeling in multi-agent environments. Amirian *et al*. [14] utilized GANs to learn multimodal pedestrian trajectory distributions. Narayanan *et al*. [15] implemented Divide-and-Conquer strategies for lane-adaptive predictions. Fang *et al*.’s [16] TPNet enhances diversity through trajectory proposal networks.

Despite the notable advancements of current data-driven vehicle trajectory prediction methods, most neural network models predominantly rely on training datasets comprising numerical data such as speed, movement direction, and position, while abstract factors like driving style can result in differing trajectories under identical external conditions. Guo *et al*. [17] comprehensively reviewed the identification and evaluation of driving characteristics and their applications in intelligent vehicles, emphasizing that driver behavior significantly impacts vehicle trajectory. Kim *et al*. [18] utilized the DeepConvLstm network and a generative-based model for driving style recognition and trajectory prediction. Chen *et al*.[19] proposed a fusion algorithm incorporating driving style and address uncertainties of vehicle dynamics to lane change trajectory prediction. Shao *et al*.[20] utilized K-means clustering to classify driving styles, but did not consider the degree of traffic congestion. Yuan *et al*.[21] develope a CNN-LSTM model for traffic conflict prediction considering the risk factors in driver merging behavior. Therefore, incorporating drivers’ behavioral characteristics and driving styles is crucial for improving prediction accuracy.

Given the aforementioned context, this study aims to develop a vehicle trajectory prediction approach that incorporates various driving styles, with the objective of filling the research gap regarding the influence of driving style discrepancies on trajectory predictions.

The main contributions of this study are as follows:

(1) The DS-TCTM model innovatively integrates driving style recognition with trajectory prediction, effectively capturing the impact of driving styles on trajectory patterns, thereby providing more accurate and personalized prediction results.
(2) The model employs a K-GMM clustering algorithm based on K-Means++, overcoming the limitations of traditional GMM models and achieving more accurate driving style recognition.
(3) The DS-TCTM model utilizes a GRU-BiGRU-BiLSTM hybrid network to effectively capture long and short-term dependencies, and employs a vertical network layer to generate personalized trajectory predictions for different driving styles.

## Architecture

**Fig 1:**
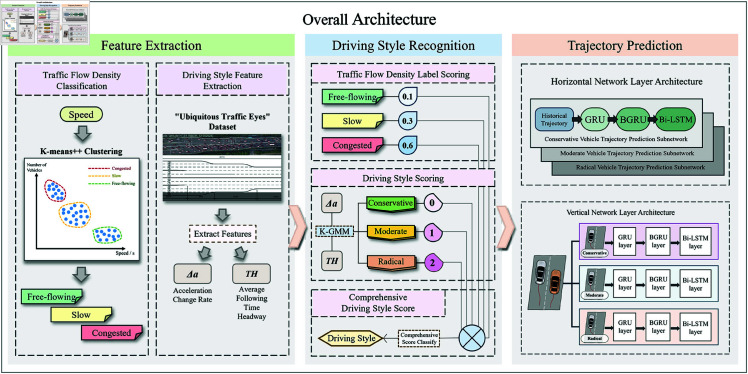
Model architecture diagram.

To enable precise short-term vehicle trajectory prediction, this study presents a driving style-based personalized prediction model. The framework incorporates three essential components: (1) Data Preprocessing Module for extracting dynamic kinematic features and traffic density metrics from raw sensor inputs; (2) Driving Style Recognition Module that transforms feature vectors into categorical style labels through machine learning techniques; (3) Personalized Trajectory Prediction Module generating individualized future trajectories conditioned on the identified driving styles. The architectural workflow is illustrated in Fig 1.

### Feature extraction module

To effectively classify driving styles, the data preprocessing module first categorizes traffic density based on vehicle speed within the road segment. Subsequently, it extracts two critical kinematic features: acceleration change rate and average time headway. Finally, the module integrates traffic density with these driving characteristics to comprehensively categorize driving styles.

#### Traffic density classification.

Traffic density significantly impacts driving styles. In high-density scenarios, drivers frequently change lanes to maintain shorter gaps for traffic fluidity. Conversely, low-density conditions allow larger gaps and stable speeds, yielding calmer driving patterns.

Since traffic density cannot be directly learned from labeled data or quantified through prior knowledge, this study employs the unsupervised K-Means++ clustering algorithm. The specific steps are as follows:

(1) Initial State Selection

Randomly select three vehicle states $h_{a},h_{b},h_{c}$, and extract their velocities $V=\{v_{a},v_{b},v_{c}\}$ as classification centers in cluster partitioning.

(2) Minimum Distance Calculation

Calculate the shortest distance between the vehicle speed at each moment and the current cluster centers:

$$d_{\min}(v_{j}^{i})=\min\left\{d_{v_{a},V_{i}},d_{v_{b},V_{i}},d_{v_{c},V_{i}}|i=1,2,\cdots ,n;j=t_{start}^{obs},\cdots ,t_{stop}^{obs}\right\}$$ (1)

where $v_{k}(k=a,b,c)$ are three classification centers in cluster partitioning, $d_{v_{a},V_{i}}=\left|v_{j}^{i}-v_{a}\right|,\;d_{v_{b},V_{i}}=\left|v_{j}^{i}-v_{b}\right|,\;d_{v_{c},V_{i}}=\left|v_{j}^{i}-v_{c}\right|$, $t_{start}^{obs}$ and $t_{stop}^{obs}$ represent the times when the vehicle enters and leaves, respectively.

(3) Sample Probability Calculation

Determine the probability for each sample point:

$$P(v_{j}^{i})=\frac{d_{\min}(v_{j}^{i}{)}^{2}}{\sum d_{\min}(v_{j}^{i}{)}^{2}}$$ (2)

Select three new samples with the maximum probabilities sequentially as updated cluster centers. Repeat the assignment and update steps until convergence is achieved, completing the algorithm.

Thus, traffic density is classified into three categories: congested, slow-moving, and free-flowing.

#### Driving style feature extraction.

Feature extraction aims to accurately identify and analyze individual driving styles. We select acceleration change rate and average time headway as key features. The acceleration change rate reveals drivers’ acceleration/deceleration habits, while the average time headway reflects their ability to maintain lane stability and safe distances.

(1) Calculation of Acceleration Change Rate

$$\Delta a(c_{i})=\frac{\sum_{t=1}^{N}(a^{t}(c_{i})-\overset{―}{a(c_{i}}){)}^{2}}{N}$$ (3)

where $\Delta a(c_{i})$ represents the acceleration change rate of vehicle *c*_*i*_, and *N* denotes the total observation time of vehicle *i* in traffic flow *d*.

(2) Calculation of Average Time Headway

$$\overset{―}{TH(c_{i})}=\frac{\overset{―}{L(c_{i})}}{\overset{―}{V(c_{i})}}$$ (4)

where $\overset{―}{TH(c_{i})}$ indicates the average time headway between vehicle *c*_*i*_ and the preceding vehicle, $\overset{―}{L(c_{i})}$ is the average following distance, and $\overset{―}{V(c_{i})}$ represents the average speed.

The unsupervised clustering analysis of traffic density combined with the extraction of Δa and *TH*, establishes the data foundation for subsequent driving style recognition and trajectory prediction.

### Driving style recognition module

To accurately analyze driving style variations under different traffic densities, we propose a traffic density-aware recognition method. This approach takes traffic density and driving style features as inputs, classifying all vehicles’ driving styles into three categories (conservative, moderate, radical) via clustering algorithms.

#### Traffic density weighting.

We categorize traffic density into three types, each assigned a weight to reflect its importance in comprehensive score calculation. During free-flow conditions, vehicles operate more freely, better reflecting driver styles, thus assigned higher weights. Under moderate density, driving becomes constrained. In congested scenarios, vehicles face severe movement restrictions requiring cautious operation, warranting the lowest weights. The specific weighting scheme is:

$$W(d)=\left\{ \begin{array}{c} 0.6,\;FreeFlow\\ 0.3,\;Slow\\ 0.1,\;Congested \end{array}\right.$$ (5)

#### Driving style scoring.

Validation via the K-Means elbow method shows that as the cluster number *k* increases, error decreases but model complexity rises. At *k* = 3, a balance between accuracy and complexity is achieved with relatively low error. Consequently, driving styles are categorized into three types: Conservative drivers prefer smooth driving patterns, maintaining larger *TH* values and lower Δa. Moderate drivers dynamically adjust their behavior across scenarios, exhibiting intermediate *TH* and Δa levels. Aggressive drivers frequently execute rapid acceleration and abrupt braking maneuvers, characterized by smaller *TH* and higher Δa. The specific scoring criteria for each driving style category are detailed below:

$$H=\{\begin{array}{ccc} Conservative, & Moderate, & Radical \end{array}\}$$ (6)

$$F(h)=\left\{ \begin{array}{c} 0,\;h=1\;Conservative\\ 1,\;h=2\;Moderate\\ 2,\;h=3\;Radical \end{array}\right.$$ (7)

#### Comprehensive driving style score calculation.

By integrating traffic density weights and driving style scores, the comprehensive driving style score is calculated as:

$$\psi (c_{i})=\sum_{h=1}^{3}W(d|c_{i}\in d)*F_{i}(h)$$ (8)

$$Class(c_{i})=\{\begin{array}{c} h|min|\Sigma_{h=1}^{3}W(d|c_{i}\in d)*F_{i}(h)-F_{i}(h)|,d=1,2,3;h=1,2,3 \end{array}\}$$ (9)

The comprehensive score effectively captures drivers’ actual characteristics across varying traffic conditions, thereby improving subsequent vehicle trajectory prediction accuracy.

### Personalized trajectory prediction module

The personalized trajectory prediction module generates short-term trajectory predictions using driving style labels and environmental features as inputs. The vehicle’s position (*x*,*y*), velocity (*v*), and acceleration (*a*) are input into personalized trajectory prediction module as input features.

**Fig 2:**
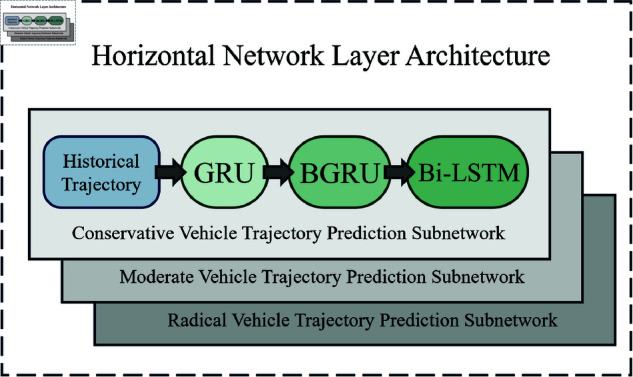
Personalized trajectory prediction model architecture.

Traditional recurrent neural networks (RNNs) struggle with long-term dependencies due to information fading. To address this, we employ a parallel bidirectional RNN architecture that enhances long-term dependency capture. The model architecture is illustrated in Fig 2.

Horizontally, the architecture implements a GRU-BiGRU-BiLSTM hybrid network, where bidirectional layers re-examine historical trajectories to improve prediction accuracy. Vertically, it conducts few-shot end-to-end learning for vehicles sharing the same driving style. Trajectory features from three distinct driving styles are fed into separate horizontal networks, enabling style-specific trajectory predictions.

#### Horizontal layer 1: GRU architecture.

The first horizontal layer employs GRU units, where each driving moment of any vehicle corresponds to a GRU cell, inputting the historical state at that moment. Update Gate Calculation:

$$z_{t}=\sigma (W_{v}*[h_{t-1},h_{j}^{i}]+b_{z})$$ (10)

where $W_{v}$ denotes the update gate’s weight matrix, *b*_*z*_ the bias vector, [] represents matrix concatenation, *h*_*t*−1_ is the hidden state vector from the previous timestep, and σ is the Swish activation function.

Reset Gate Calculation:

$$r_{t}=\sigma (W_{r}*[h_{t-1},h_{j}^{i}]+b_{r})$$ (11)

where *W*_*r*_ is the reset gate’s weight matrix and *b*_*r*_ the corresponding bias vector.

**Fig 3:**
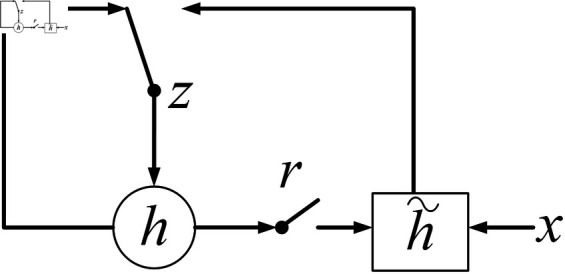
Basic GRU gate structure.

Candidate State Generation:

$$h_{t}=\tanh(W_{h}*[r_{t}*h_{t-1},z_{t}]+b_{h})$$ (12)

here, *W*_*h*_ represents the hidden state weight parameters, *b*_*h*_ the bias vector, and tanh the hyperbolic tangent function. The basic GRU gate structure is illustrated in Fig 3.

#### Horizontal layer 2: BGRU architecture.

**Fig 4:**
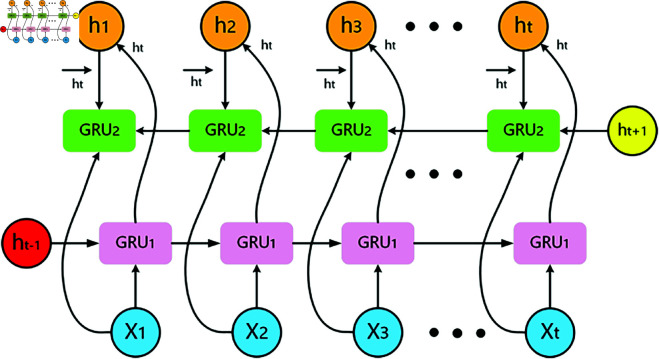
BGRU network architecture.

The second horizontal layer employs Bidirectional GRU (BiGRU), comprising an input layer, forward hidden layer, backward hidden layer, and output layer. Data flows from the input layer to both directional hidden layers, where two opposing GRU networks jointly determine the output. The BGRU network architecture is illustrated in Fig 4. The mathematical formulation is:

$$h_{m}=GRU_{1}(x_{t},h_{m-1})\;h_{n}=GRU_{2}(x_{t},h_{n+1})\;h_{t}=f(W_{h_{m}}*h_{m}+W_{h_{n}}*h_{n}+b_{t})$$ (13)

where *x*_*t*_ is the input vector at timestep *t*, *h*_*m*_ and *h*_*n*_ denote the forward and backward hidden states respectively, $W_{h_{m}}$ and $W_{h_{n}}$ are their corresponding weight matrices, and *b*_*t*_ represents the bias term.

#### Horizontal layer 3: BiLSTM architecture.

**Fig 5:**
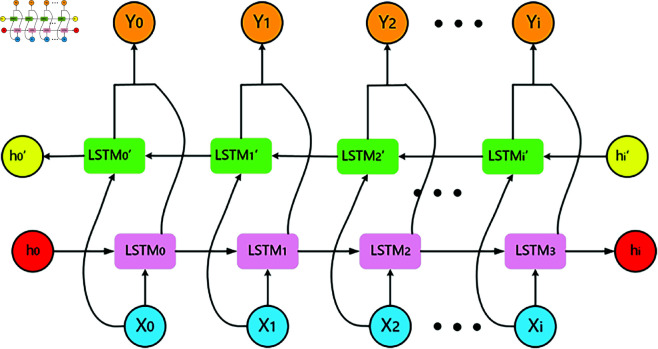
BiLSTM network architecture.

The third horizontal layer utilizes BiLSTM, processing the same input sequence through forward and backward LSTM hidden layers, with both outputs contributing to the final layer. Compared to unidirectional LSTM, BiLSTM’s bidirectional structure enhances data utilization efficiency, overcomes traditional LSTM’s limitations in temporal data processing, and exhibits stronger robustness and generalization capabilities. The BiLSTM network architecture is illustrated in Fig 5.

Each timestep corresponds to an LSTM gate, where the vehicle’s historical state $h_{i}^{t}$ serves as the input signal *x*_*t*_. The BiLSTM operations are defined as:

$$h_{t}=LSTM(x_{t},h_{t-1})\;h_{t}^{\prime }=LSTM(x_{t},h_{t-1}^{\prime })\;y_{t}=W_{h}*h_{t}+W_{h}^{\prime }*h_{t}^{\prime }+b_{y}$$ (14)

here *h*_*t*_ and $h_{t}^{\prime }$ represent forward and backward LSTM outputs at timestep *t*, *W*_*h*_ and $W_{h}^{\prime }$ are their respective weight matrices, and *b*_*y*_ denotes the bias term.

#### Vertical network layer architecture.

**Fig 6:**
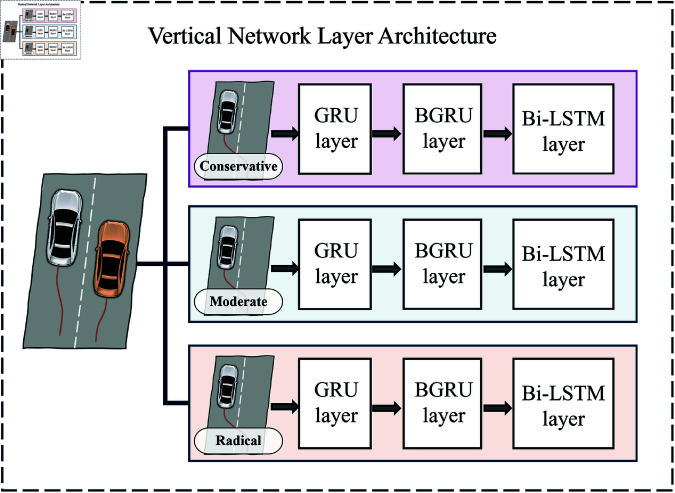
Vertical network layer architecture diagram.

To comprehensively consider driving style impacts on trajectories, we feed style-specific trajectories into three independent horizontal networks for few-shot end-to-end deep learning. The vertical hierarchy comprises three parallel identical network architectures, integrating GRU, BGRU, and BiLSTM advantages to achieve precise short-term trajectory predictions. By training three distinct prediction models with different driving style data, this design fully utilizes existing data, enhances few-shot learning capability, and significantly improves prediction accuracy for varied driving styles. The Vertical network layer architecture is illustrated in Fig 6.

## Experimental design and analysis

### Dataset selection

**Fig 7:**
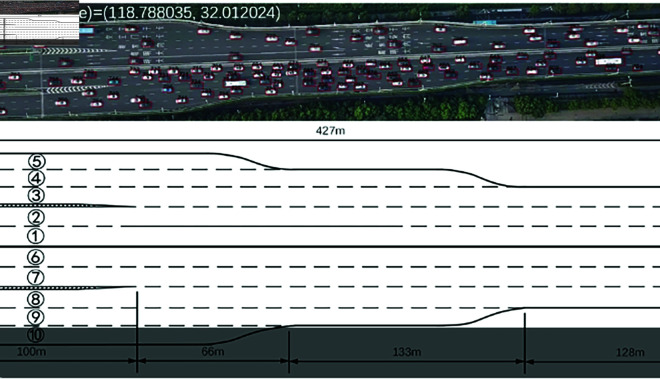
Ubiquitous traffic eyes dataset.

This study utilizes the “Ubiquitous Traffic Eyes” dataset [22] and the highD dataset, and the details of these two datasets are described as follows:

(1) The "Ubiquitous Traffic Eyes" dataset encompasses diverse traffic scenarios and driving styles across various weather conditions, time periods, and geographical locations. The dataset contains trajectory records from over 100,000 vehicles, including critical parameters such as position, velocity, acceleration, steering angle, and timestamps, providing a robust foundation for trajectory prediction and driving style recognition. Fig 7 shows the scene environment of the Ubiquitous traffic eyes dataset.

(2) The highD dataset is a new dataset of naturalistic vehicle trajectories recorded on German highways. Using a drone, typical limitations of established traffic data collection methods such as occlusions are overcome by the aerial perspective. Traffic was recorded at six different locations and includes more than 110 500 vehicles. Each vehicle’s trajectory, including vehicle type, size and manoeuvres, is automatically extracted. Using state-of-the-art computer vision algorithms, the positioning error is typically less than ten centimeters. Although the dataset was created for the safety validation of highly automated vehicles, it is also suitable for many other tasks such as the analysis of traffic patterns or the parameterization of driver models.

### Parameter setting

The backbone networks of the models employed in this paper are GRU, BGRU, and Bi-LSTM, with GRU and BGRU comprising 150 nodes each, and Bi-LSTM consisting of 200 nodes.The optimizer used is Adagrad. Batch_size is set to 8, the number of epochs to 150, and the initial learning rate to 0.001. The experiment develops a neural network tra-jectory prediction model using Python 3.6 and TensorFlow 1.4.0, with the hardware envi-ronment consisting of a PC with 11th Gen Intel(R) Core(TM) i7-11800H @2.30 GHz and NVIDIA GeForce RTX 3060, running Windows 10.

### Data preprocessing

In order to eliminate GPS measurement errors and occasional anomalies, a three-step denoising process was adopted in the data preprocessing stage:

(1) Outlier detection and rejection

The mean and standard σ deviation of velocity *v* and acceleration *a* in each trajectory are calculated, and the elimination is satisfied The mean μ and standard deviation σ of velocity *v* and acceleration *a* in each trajectory are calculated, and the time point satisfying

$$v>\mu_{v}+3\sigma_{v}\;or\;a>\mu_{v}+3\sigma_{v}$$ (15)

is excluded to exclude obvious measurement jumps.

(2) Savitzky–Golay smoothing filtering

Apply a Savitzky-Golay filter with a window length of 5 and a quadratic polynomial to the *x* and *y* coordinates of the trajectory sequence after removing outliers, further suppressing high-frequency jitter and ensuring the smoothness of the trajectory.

(3) Linear interpolation complementation

For short-term missing data (≤3 frames) caused by outlier removal or filtering, one-dimensional linear interpolation is used to fill in the gaps, ensuring the continuity of the input sequence; if the missing data exceeds 3 frames, the corresponding sliding window is discarded.

This study utilizes the publicly available Ubiquitous Traffic Eyes dataset and highD dataset. Ubiquitous Traffic Eyes collected a total of 1,810,742 continuous vehicle trajectories, while highD is stored as 60 files, one of which has 300,000-400,000 continuous vehicle trajectories. The data is divided into training, validation, and test sets in an 8:1:1 ratio.

### Evaluation metrics and baseline models

#### Evaluation metrics.

This experiment employs Root Mean Square Error (RMSE), Negative Log-Likelihood (NLL), Mean Absolute Error(MAE) and Final displacement error (FDE) to assess model performance. RMSE quantifies the deviation between predicted and ground-truth trajectories, NLL evaluates prediction accuracy of vehicle action types, MAE represents the average value of the absolute error between the predicted and observed values, and FDE measured the deviation between the predicted endpoint and the true endpoint in the last step of the prediction. Each model group undergoes 100 experimental trials, with metric means recorded. The calculations are defined as:

$$RMSE=\sqrt{\frac{1}{n}\sum_{i=1,2,\ldots ,n}\left({\left({\hat{x}}_{i}-x_{i}\right)}^{2}+{\left({\hat{y}}_{i}-y_{i}\right)}^{2}\right)}$$ (16)

$$NLL=loss_{nll}(L_{true},L_{pre})$$ (17)

$$MAE=\frac{1}{n}\sum_{i=1,2,\ldots ,n}(|{\hat{x}}_{i}-x_{i}|+|{\hat{y}}_{i}-y_{i}|)$$ (18)

$$FDE=\sqrt{{\left({\hat{x}}_{end}-x_{end}\right)}^{2}+{\left({\hat{y}}_{end}-y_{end}\right)}^{2}}$$ (19)

where ${\hat{x}}_{i}$ and ${\hat{y}}_{i}$ denote the predicted lateral and longitudinal positions, *loss*_*nll*_ represents the negative log-likelihood loss function, *L*_*true*_ and *L*_*pre*_ denote the ground-truth trajectories and the model-predicted trajectories respectively, ${\hat{x}}_{end}$ and ${\hat{y}}_{end}$ denote the longitude and latitude of the predicted location of the end position, *x*_*end*_ and *y*_*end*_ denote the longitude and latitude of the true location of the end position.

#### Baseline models.

This paper adopts LSTM [23], Social-LSTM [24], CS-LSTM [25], SV-LSTM [26] (which adds vehicle speed information to the Social-LSTM structure), TCTM [27] (without considering driving styles), STA-LSTM [28] and LSTM GAN [29] as baseline models for comparing prediction accuracy and computational efficiency.

### Driving style clustering model comparison

This experiment, conducted on the Ubiquitous Traffic Eyes Dataset, classifies driving styles by extracting features such as average time headway (*TH*) and acceleration change rate (Δa ), comparing the performance between standard GMM and improved K-GMM models.

**Fig 8:**
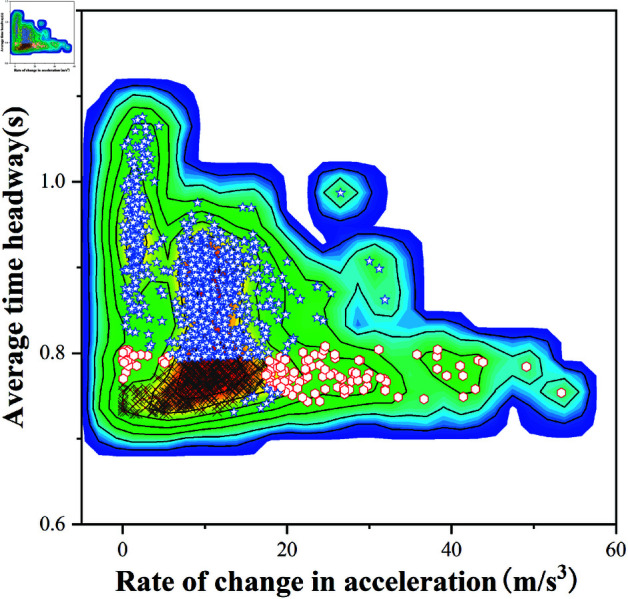
Contour plot of standard GMM clustering.

Contour plot analysis reveals similar overall shapes between Fig 8 (GMM) and Fig 10 (K-GMM). The high-density region in Fig 8 concentrates in the central-lower area with smooth color transitions and uniform data point distribution. In contrast, Fig 10 exhibits slightly dispersed color distribution at boundaries, indicating tighter clustering in K-GMM.

**Fig 9:**
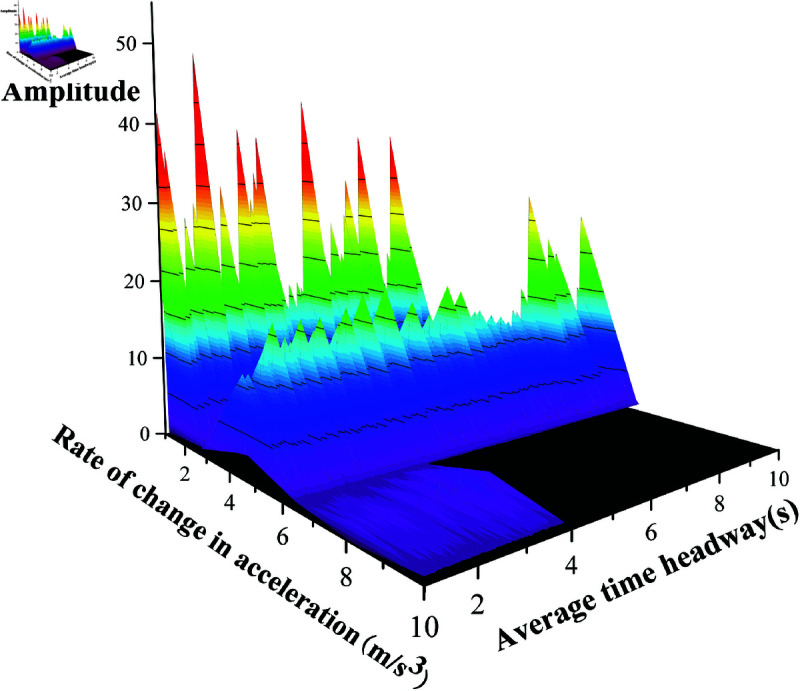
Peak distribution of standard GMM clustering.

3D histogram comparisons (Fig 9 vs. Fig 11) show analogous amplitude distributions. Fig 9 displays scattered high-amplitude peaks, particularly in large Δa regions, while Fig 11 demonstrates more concentrated peaks. The smoother color gradients in Fig 9 contrast with steeper transitions in Fig 11, suggesting K-GMM’s enhanced discriminative capability in specific regions.

**Fig 10:**
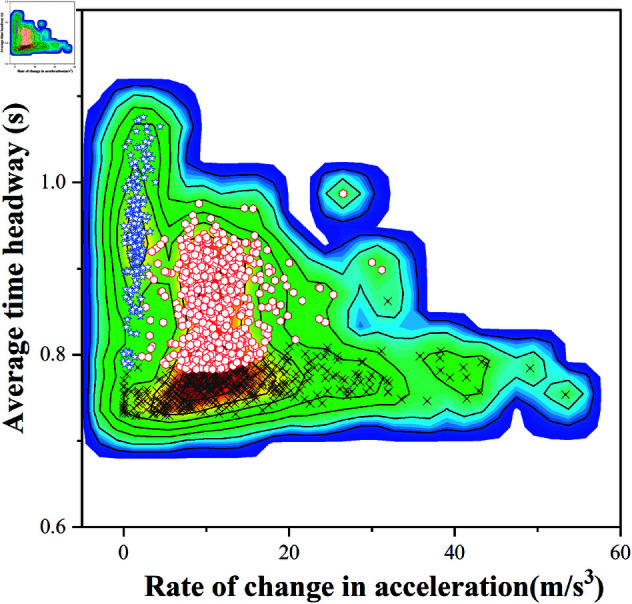
Contour plot of K-GMM clustering.

**Fig 11:**
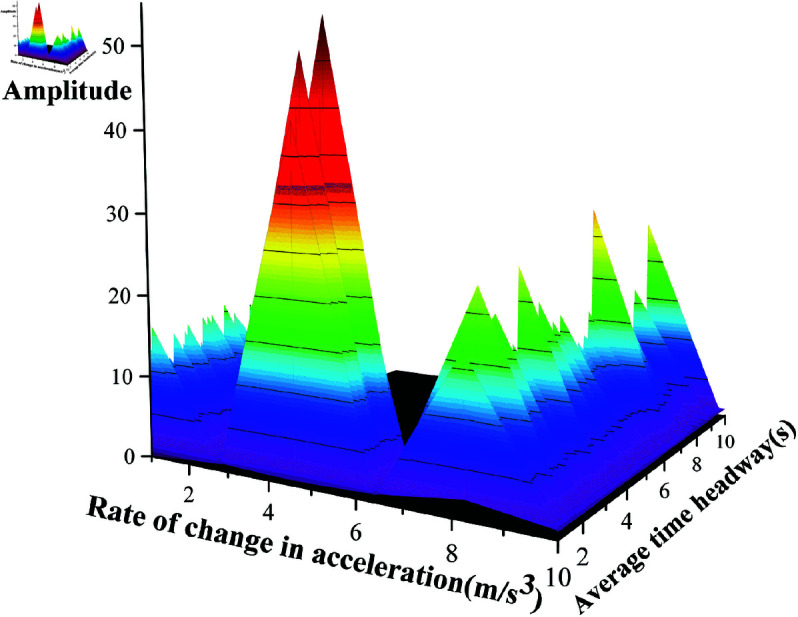
Peak distribution of K-GMM clustering.

Quantitatively, K-GMM achieves tighter cluster compactness in high-density and high-amplitude regions, effectively grouping similar driving styles. Comparative analyses of Figs 8, 9, 10, and 11 validate K-GMM’s superior clustering precision and amplitude differentiation over standard GMM, confirming its effectiveness on this dataset.

### Fundamental performance analysis

This section evaluates the core predictive capabilities of DS-TCTM against baseline models through four key metrics: RMSE, NLL, MAE and FDE.

#### Ubiquitous traffic eyes dataset.

Table 1 shows the comparison of RMSE in ubiquitous traffic eyes. All models exhibit increasing RMSE with longer prediction horizons. DS-TCTM achieves significantly lower RMSE across all timepoints, demonstrating superior trajectory prediction accuracy.

**Table 1:** Comparison of RMSE in ubiquitous traffic eyes.

Model	RMSE at different times in the future				
	1s-sec	2-sec	3-sec	4s-sec	5-sec
V-LSTM	0.68	1.76	3.21	4.82	6.87
S-LSTM	0.63	1.43	2.63	4.21	6.18
SV-LSTM	0.62	1.41	2.44	3.87	5.73
CS-LSTM	0.65	1.34	2.40	3.56	4.54
TCTM	0.21	0.85	1.88	2.99	5.39
STA-LSTM	0.41	0.95	1.78	**2.63**	**3.49**
LSTM GAN	0.32	0.68	1.74	2.99	4.87
DS-TCTM	**0.11**	**0.64**	**1.45**	**2.63**	4.46

Table 2 shows the comparison of NLL in ubiquitous traffic eyes. Like RMSE trends, NLL values increase with prediction duration. DS-TCTM maintains a lower NLL at most horizons, indicating better probabilistic distribution fitting.

**Table 2:** Comparison of NLL in ubiquitous traffic eyes.

Model	NLL at different times in the future				
	1s-sec	2-sec	3-sec	4s-sec	5-sec
V-LSTM	1.57	3.07	3.92	3.56	5.28
S-LSTM	0.72	2.11	**2.84**	3.42	3.95
SV-LSTM	0.64	2.07	2.88	3.49	3.92
CS-LSTM	0.62	2.09	3.04	3.65	4.01
TCTM	0.77	2.24	2.96	3.54	3.99
STA-LSTM	1.51	2.62	3.74	4.02	4.51
LSTM GAN	1.25	**2.02**	3.14	4.01	4.98
DS-TCTM	**0.51**	2.31	2.88	**3.22**	**3.89**

Table 3 shows the comparison of MAE in ubiquitous traffic eyes. Like RMSE and NLL trends, MAE values increase with prediction duration. Compared to other baseline models, DS-TCTM maintains a lower MAE.

**Table 3:** Comparison of MAE in ubiquitous traffic eyes.

Model	MAE at different times in the future				
	1s-sec	2-sec	3-sec	4s-sec	5-sec
V-LSTM	0.312	0.338	0.401	0.465	0.590
S-LSTM	0.295	0.324	0.385	0.439	0.550
SV-LSTM	0.278	0.307	0.362	0.362	0.520
CS-LSTM	0.259	0.291	0.345	0.395	0.490
TCTM	0.238	0.273	0.321	0.370	0.460
STA-LSTM	0.220	0.256	0.304	0.353	0.435
LSTM GAN	0.215	0.248	0.321	0.367	0.415
DS-TCTM	**0.203**	**0.240**	**0.290**	**0.340**	**0.410**

Table 4 shows the comparison of FDE in ubiquitous traffic eyes. FDE is used to evaluate the effectiveness of control algorithms, and DS-TCTM has smaller FDE instructions to be able to predict vehicle trajectories more accurately.

**Table 4:** Comparison of FDE in ubiquitous traffic eyes.

Model	FDE at different times in the future				
	1s-sec	2-sec	3-sec	4s-sec	5-sec
V-LSTM	3.31	3.33	4.45	5.86	6.99
S-LSTM	3.29	3.26	3.98	4.73	5.65
SV-LSTM	3.17	3.32	3.66	3.92	4.57
CS-LSTM	3.05	3.30	3.35	3.69	4.29
TCTM	3.28	3.22	3.32	3.34	3.46
STA-LSTM	3.12	3.16	3.36	3.63	3.84
LSTM GAN	3.21	3.33	3.48	3.53	3.64
DS-TCTM	**2.90**	**3.14**	**3.29**	**3.30**	**3.41**

#### HighD dataset.

Table 5 shows the comparison of RMSE in HighD. As in the Ubiquitous Traffic Eyes dataset, all models exhibit increasing RMSE with longer prediction horizons in the highD dataset. DS-TCTM achieves significantly lower RMSE across all timepoints, demonstrating superior trajectory prediction accuracy.

**Table 5:** Comparison of RMSE in HighD.

Model	RMSE at different times in the future				
	1s-sec	2-sec	3-sec	4s-sec	5-sec
V-LSTM	0.65	1.72	3.61	4.22	5.97
S-LSTM	0.79	1.23	2.33	3.92	5.18
SV-LSTM	0.69	1.51	2.24	3.47	4.93
CS-LSTM	0.55	1.44	2.30	3.26	3.94
TCTM	0.15	0.65	1.58	2.45	3.15
STA-LSTM	0.45	0.65	1.38	2.58	3.29
LSTM GAN	0.32	0.56	1.29	2.46	3.01
D S-TCTM	**0.09**	**0.36**	**1.25**	**2.03**	**2.76**

Table 6 shows the comparison of NLL in HighD. As in the Ubiquitous Traffic Eyes dataset, NLL values increase with prediction duration and DS-TCTM maintains a lower NLL at most horizons, indicating better probabilistic distribution fitting.

**Table 6:** Comparison of NLL in HighD.

Model	NLL at different times in the future				
	1s-sec	2-sec	3-sec	4s-sec	5-sec
V-LSTM	1.57	3.07	3.92	4.56	5.28
S-LSTM	0.72	2.11	**2.84**	3.42	3.95
SV-LSTM	0.64	2.07	2.88	3.49	3.92
CS-LSTM	0.62	**2.09**	3.04	3.65	4.01
TCTM	0.77	2.24	2.96	3.54	3.99
STA-LSTM	1.51	2.62	3.74	4.02	4.51
LSTM GAN	1.82	2.66	3.85	4.62	5.12
DS-TCTM	**0.51**	2.31	2.88	**3.22**	**3.89**

Table 7 shows the comparison of MAE in HighD. Compared with other baseline models, DS-TCTM maintains a lower MAE at each time on the highD dataset.

**Table 7:** Comparison of MAE in ubiquitous traffic eyes.

Model	MAE at different times in the future				
	1s-sec	2-sec	3-sec	4s-sec	5-sec
V-LSTM	0.278	0.315	0.372	0.429	0.517
S-LSTM	0.262	0.298	0.355	0.403	0.480
SV-LSTM	0.245	0.281	0.330	0.378	0.445
CS-LSTM	0.228	0.264	0.310	0.357	0.410
TCTM	0.210	0.247	0.290	0.336	0.390
STA-LSTM	0.195	0.232	0.275	0.320	0.370
LSTM GAN	0.184	0.205	0.257	0.301	0.368
DS-TCTM	**0.142**	**0.180**	**0.225**	**0.270**	**0.310**

Table 8 shows the comparison of FDE in HighD. As in the Ubiquitous Traffic Eyes dataset, DS-TCTM has smaller FDE in the highD dataset.

**Table 8:** Comparison of FDE in HighD.

Model	FDE at different times in the future				
	1s-sec	2-sec	3-sec	4s-sec	5-sec
V-LSTM	5.24	5.57	6.60	7.94	8.68
S-LSTM	5.11	5.03	6.20	6.80	7.81
SV-LSTM	4.92	5.57	5.85	5.75	6.36
CS-LSTM	4.89	5.20	5.36	5.65	6.16
TCTM	5.34	5.03	5.21	5.27	5.44
STA-LSTM	5.27	5.01	5.37	5.68	5.58
LSTM GAN	5.27	5.15	5.24	5.78	5.90
DS-TCTM	**4.55**	**4.84**	**5.08**	**5.24**	**5.38**

### Computational efficiency

This experiment compares the computational efficiency between baseline models and DS-TCTM by measuring processing time per vehicle trajectory.

Table 9 demonstrates the processing time required by each model. V-LSTM achieves the shortest latency, while CS-LSTM exhibits the poorest efficiency. From the previous RMSE and NLL comparisons, the DS-TCTM model demonstrates superior prediction accuracy. Although not the most computationally efficient, DS-TCTM achieves an optimal balance between prediction accuracy and processing speed. In contrast, while V-LSTM has the highest computational efficiency, its prediction accuracy (as indicated by RMSE and NLL values) is inferior to DS-TCTM.

**Table 9:** Comparison of calculation efficiency.

Model	V-LSTM	S-LSTM	SV-LSTM	CS-LSTM	TCTM	STA-LSTM	LSTM GAN	DS-TCTM
Time(ms)	**10.7**	15.8	16.3	19.8	12.6	25.4	55.6	13.4

## Conclusions

(1) Model Innovation

Addressing vehicle trajectory prediction challenges, this study proposes the DS-TCTM model that integrates traffic density and driving style impacts for personalized prediction. The architecture combines multi-scale temporal modeling with driving style characterization through an enhanced K-GMM clustering approach (K-Means++ based), effectively resolving sample segmentation issues while capturing long-term dependencies.

(2) Performance Superiority

Experimental results confirm DS-TCTM’s superior performance, with RMSE and NLL values maintained below 4.46 and 3.89 respectively. Compared to conventional LSTM models, it achieves 35.8% error reduction, demonstrating particular strength in long-term predictions.

(3) Future Directions

The current unsupervised clustering approach with manual labeling provides initial style differentiation, yet algorithmic refinement of the K-GMM-based scoring system presents a key avenue for enhancing model precision in future work, particularly for deployment in connected vehicle applications.
